# Development, validation and visualization of a web-based nomogram for predicting risk of new-onset diabetes after percutaneous coronary intervention

**DOI:** 10.1038/s41598-024-64430-9

**Published:** 2024-06-13

**Authors:** Mengmeng Zhu, Yiwen Li, Wenting Wang, Yanfei Liu, Tiejun Tong, Yue Liu

**Affiliations:** 1 National Clinical Research Center for TCM Cardiology, Xiyuan Hospital, China Academy of Chinese Medical Sciences, No.1 of Xiyuan Caochang, Haidian District, Beijing, 100091 China; 2 Cardiovascular Disease Group, China Center for Evidence-Based Medicine of TCM, China Academy of Chinese Medical Sciences, Beijing, China; 3 Beijing Key Laboratory of Traditional Chinese Medicine Basic Research on Prevention and Treatment for Major Diseases, Experimental Research Center, China Academy of Chinese Medical Sciences, Beijing, China; 4 The Second Department of Geriatrics, Xiyuan Hospital, China Academy of Chinese Medical Sciences, Beijing, China; 5 Department of Mathematics, Hong Kong Baptist University, Kowloon Tong, Hong Kong, SAR China

**Keywords:** Cardiovascular diseases, Percutaneous coronary intervention, New-onset diabetes, Risk factors, Predictive model, Cardiology, Endocrinology, Risk factors

## Abstract

Simple and practical tools for screening high-risk new-onset diabetes after percutaneous coronary intervention (PCI) (NODAP) are urgently needed to improve post-PCI prognosis. We aimed to evaluate the risk factors for NODAP and develop an online prediction tool using conventional variables based on a multicenter database. China evidence-based Chinese medicine database consisted of 249, 987 patients from 4 hospitals in mainland China. Patients ≥ 18 years with implanted coronary stents for acute coronary syndromes and did not have diabetes before PCI were enrolled in this study. According to the occurrence of new-onset diabetes mellitus after PCI, the patients were divided into NODAP and Non-NODAP. After least absolute shrinkage and selection operator regression and logistic regression, the model features were selected and then the nomogram was developed and plotted. Model performance was evaluated by the receiver operating characteristic curve, calibration curve, Hosmer–Lemeshow test and decision curve analysis. The nomogram was also externally validated at a different hospital. Subsequently, we developed an online visualization tool and a corresponding risk stratification system to predict the risk of developing NODAP after PCI based on the model. A total of 2698 patients after PCI (1255 NODAP and 1443 non-NODAP) were included in the final analysis based on the multicenter database. Five predictors were identified after screening: fasting plasma glucose, low-density lipoprotein cholesterol, hypertension, family history of diabetes and use of diuretics. And then we developed a web-based nomogram (https://mr.cscps.com.cn/wscoringtool/index.html) incorporating the above conventional factors for predicting patients at high risk for NODAP. The nomogram showed good discrimination, calibration and clinical utility and could accurately stratify patients into different NODAP risks. We developed a simple and practical web-based nomogram based on multicenter database to screen for NODAP risk, which can assist clinicians in accurately identifying patients at high risk of NODAP and developing post-PCI management strategies to improved patient prognosis.

## Introduction

Acute coronary syndrome (ACS) is a severe condition that poses a significant threat to human health and life. Percutaneous coronary intervention (PCI) is currently the primary therapeutic approach used to restore blood perfusion and salvage the ischemic myocardium as soon as possible after the onset of ACS^1^. Among patients undergoing PCI, more than 20% are diagnosed with diabetes mellitus^2^. Extensive evidence suggests that pre-existing type 2 diabetes can elevate the risk of major adverse cardiac events (MACE), all-cause mortality, and restenosis post-PCI, resulting in unfavorable outcomes^3–5^. However, the incidence of new-onset diabetes after PCI (NODAP) at 3.4 ± 1.9 years after PCI can reach 11.8%, which is an issue that has not received sufficient clinical attention^6^. A previous study examining the postoperative outcomes of 3599 patients who underwent PCI revealed that newly diagnosed diabetes is significantly associated with increased 3-year mortality and MACE post-PCI compared to patients without diabetes^7^. Therefore, early assessment and accurate prediction of the risk of NODAP, as well as timely control of risk factors, are essential for minimizing adverse events following PCI.

Existing models of new-onset diabetes in patients with coronary heart disease have some capability in identifying patients with risk of NODAP^8^. However, patients undergoing PCI in clinical practice often have more severe manifestations and more complex postoperative drug regimens. The influence of type 2 diabetes risk factors, such as high triglyceride (TG) level and high fasting plasma glucose (FPG) level, and the effect of statin drugs on the incidence of NODAP remain unclear^6^. To date, there is no dedicated prediction model for the risk of NODAP. A nomogram is a straightforward statistical visualization tool, has gained widespread use in recent years for predicting disease occurrence, progression, and prognosis^9^. In this study, we utilized patient data from four clinical centers across three provinces and cities in China. Through selection of variables influencing the risk of NODAP, we developed a NODAP nomogram and conducted internal and external validation for this model. This study aimed to facilitate early clinical screenings of high-risk NODAP populations and to optimize postoperative management of patients undergoing PCI to assist with clinical decision-making.

## Methods

### Data source

Data in this study were derived from China Evidence-based Chinese Medicine (CECM) database, hosted by China Center for Evidence Based Traditional Chinese Medicine and co-organized by Beijing Econ Network Technology Co., Ltd. The database collected data from 6, 465, 493 electronic medical records of 249, 987 outpatients or inpatients at Xiyuan Hospital of China Academy of Traditional Chinese Medicine (Beijing, China), Beijing Hospital of Traditional Chinese Medicine (Beijing, China), Jiangsu Hospital of Traditional Chinese Medicine (Nanjing, Jiangsu, China), and the First Affiliated Hospital of Henan University of Traditional Chinese Medicine (Zhengzhou, Henan, China) from 2007 to 2022. The medical record data included demographic information, diagnoses according to International Classification of Diseases 10th Revision (ICD-10) disease codes, prescription records, laboratory tests, and medical records. And all identifying patient information within the database was removed. Clinical data contains a large amount of noise, outliers, etc. Using Extract-Transform-Loa (ETL) tool Kettle, combined with the platform's custom function and natural language processing (NLP) and other technologies for data conversion, cleaning and extraction to complete the deletion or correction of bad data as well as data de-weighting and categorization. Different hospitals include different data types and indicator units, which may lead to bias. Structured data are formed through the processing of terminology standardization, coding standardization, interface standardization, data exchange standardization, etc. And a standardized and normalized real-world database is finally established. The detailed protocol of the study has been published previously^10^.

### Data processing

All patients undergoing PCI for ACS were retrieved from the database using techniques such as NLP and relation extraction, and then the final study population was screened according to inclusion and exclusion criteria. Based on prior literature studies^6,11–13^, we identified 28 candidate predictor variables, for the definitions of which see Table S1. Extract the results of all the following variables included in the study population: gender, age, systolic blood pressure (SBP), diastolic blood pressure (DBP), heart rate (HR), glycated hemoglobin (HbA1c), fasting plasma glucose (FPG), total cholesterol (TC), triglyceride (TG), low-density lipoprotein cholesterol (LDL-C), high-density lipoprotein cholesterol (HDL-C), serum creatinine (Scr), history of smoking, history of alcohol intake, history of hypertension, history of hyperlipemia, family history of diabetes and prescription of drugs.

The missing rates of the variables were counted (Table S2), and one of the variables with a missing rate of more than 30% was HbA1c, which was directly excluded because of its high level of missingness. The missing rate of 0% to 30% were SBP, DBP, HR, FPG, TC, TG, LDL-C, HDL-C and Scr, which were filled by multiple imputation. Multiple imputation method based on Bayesian framework was applied to the above dataset to estimate the missing values by using regression model through multiple iterations, 10 update operations were performed on all the missing values, and the final complete data was obtained after 5 iterations. The final number of variables was determined to be 27.

### Ethics statement

The study was registered at the Chinese Clinical Trial Registry (ChiCTR2100047241). This study was approved by the Ethics Committee of the Xiyuan Hospital of China Academy of Chinese Medical Sciences (approval number 2021XL007-2), and all methods were carried out in accordance with relevant guidelines and regulations. Requirement for informed consent was waived due to the retrospective nature of the study.

### Study population

The hospital medical records from 2007 to 2022 were included for retrospective analysis. The inclusion criteria were as follows: (1) age ≥ 18 years; (2) coronary stent implantation for ACS. The exclusion criteria were as follows: (1) severe heart diseases such as congenital heart disease, severe cardiac insufficiency (cardiac function Grade III or above), or severe valvular disease; (2) previous surgical procedures such as cardiac bypass surgery, pacemaker implantation, valve replacement surgery, other cardiac surgeries, pancreatectomy, or organ transplant surgery; (3) diseases that impact glucose metabolism such as hyperthyroidism, acromegaly, and Cushing's syndrome; (4) diagnosis of diabetes prior to PCI; (5) follow-up duration of < 1-year post-PCI.

### Study endpoint

The study endpoint was defined as the occurrence of NODAP. NODAP is defined as any of the following conditions that were not present prior to PCI but are present after PCI^6,7^: (1) FPG level ≥ 7.0 mmol/L, (2) random blood glucose level ≥ 11.1 mmol/L; (3) HbA1c level ≥ 6.5%; (4) diagnosis of type 2 diabetes using ICD-10 codes E11.001–E11.901; (5) antidiabetic drug therapy.

### Statistical analysis

Data from the Xiyuan Hospital of the China Academy of Chinese Medical Sciences, Beijing Hospital of Traditional Chinese Medicine, and Jiangsu Province Hospital of Traditional Chinese Medicine were included in the training cohort, and the above three centers were considered as independent and identically distributed queues^6,11–13^. The internal validation cohort was drawn from one-third of the training cohort by a completely blinded, random sampling approach. Data from the First Affiliated Hospital of Henan University of Chinese Medicine were included in the external validation cohort. The training cohort was used to explore potential factors influencing NODAP and develop a predictive model, whereas the internal and external validation cohorts were used to evaluate the performance of the predictive model.

For measurement data conforming to a normal distribution, data are expressed as mean ± standard deviation, and comparisons between groups were performed using independent sample t-tests. For data not adhering to a normal distribution, data are expressed as M [Q1, Q3], and comparisons between groups were performed using the Mann–Whitney U test. Count data are expressed as frequency (%), and comparisons between groups were performed using the χ^2^ test. The Kaplan–Meier method was used to calculate and plot the cumulative incidence curve for NODAP.

After standardizing and normalizing the data, transform it into a form suitable for machine learning and start model training. In the training cohort, least absolute shrinkage and selection operator (LASSO) regression analysis was used to reduce the dimensions of variables. After 10 cross-validations were performed, predictive factors were selected. The selected predictive factors were further refined through multivariate logistic two-way stepwise regression analysis with a test level of alpha = 0.05 to select statistically significant factors as the final risk factors for NODAP, and the results were expressed as the odds ratio (OR) and 95% CI. The risk factors were then analyzed for covariance using the tolerance and variance expansion factor, which satisfied the tolerance > 0.1 and variance inflation factor < 10, indicating that there was no covariance relationship among the variables. A clinical prediction model was established, and a nomogram was plotted. The receiver operating characteristic (ROC) curve was used to evaluate the discrimination of the model. The calibration curves and Hosmer–Lemeshow test were used to assess the precision of the model, and a *p* value of the Hosmer–Lemeshow test greater than 0.05 indicates that a model has high goodness of it. Decision curve analysis (DCA) was used to evaluate the clinical benefit of the model.

All study subjects in the training cohort were risk-scored using the model, and based on the predicted scores, the training population was categorized into low, moderate, and high-risk groups according to the same sample size percentages, and their corresponding risk scores were used as the risk stratification cutoffs for the prediction model. All study subjects in the entire cohort were then risk scored and included into different risk stratifications. Kaplan–Meier curves were used to observe the differences in cumulative incidence rates for all study subjects under different risk stratifications.

All data analyses in this study were performed using Python Programming Language 3.8.5 (https://www.python.org/downloads/release/python-385/). The “statsmodels.imputation” package was utilized for multiple imputations of missing data. We used “scipy.stats” and “pandas” for statistical analysis and consistency testing. For cumulative incidence curve plotting, we used “matplotlib.pyplot” and the “lifelines” package. For predictive factor selection, construction, and nomogram plotting, we used “sklearn.linear_model,” “matplotlib.pyplot,” “statsmodels,” “matplotlib,” and the “zepid.graphics” packages. All tests were two-sided with a significance level of α = 0.05, and a *p* value < 0.05 was considered statistically significant. All analyses were conducted according to the guidelines of the Transparent Reporting of Multivariate Predictive Models for Individual Prognosis or Diagnosis (TRIPOD)^14^.

## Results

### Cohort characteristics

**Figure 1:**
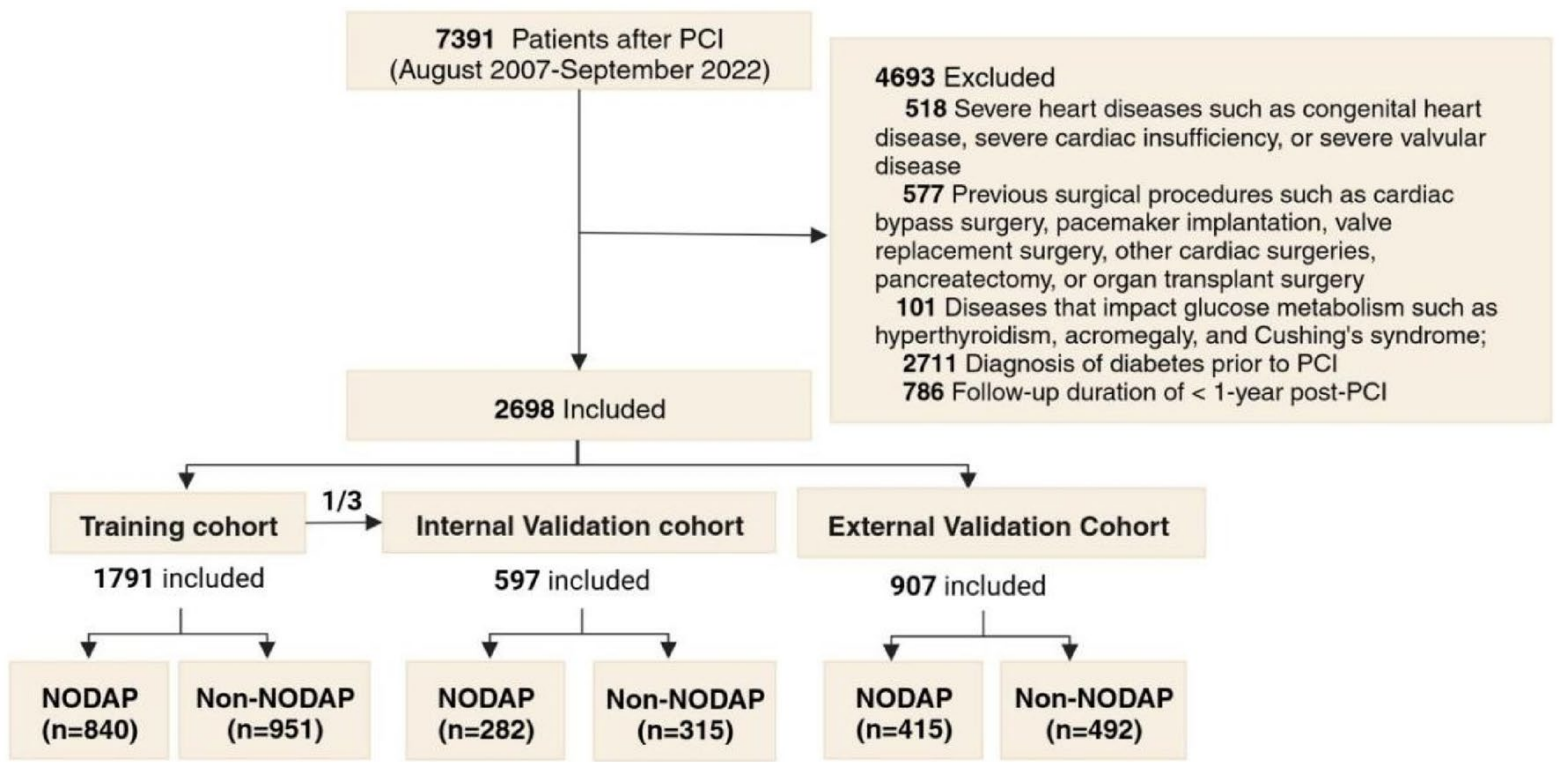
Flow chart of study cohort establishment. PCI, percutaneous coronary intervention; NODAP, New-Onset Diabetes After percutaneous coronary intervention.

A total of 7391 post-PCI patients were obtained based on database screening, and a total of 2698 patients were included in this study according to the inclusion and exclusion criteria (1255 NODAP patients and 1443 non-NODAP patients), including 1791 in the training cohort and 907 in the external validation cohort, and then 597 patients were randomly selected from the training cohort into the internal validation cohort. The establishment process of the study cohort is shown in Fig. 1.

Table 1 outlines the baseline characteristics of the training, internal validation, and external validation cohorts. The baseline characteristics of the internal validation cohort were consistent with those of the training cohort. The external validation cohort exhibited lower age ranges, decreased total TC levels, increased LDL-C levels, increased use of statin and psychotropic drugs, and decreased use of angiotensin receptor blocker (ARB), β-blocker, and calcium channel blocker (CCB), as well as comorbid hypertension and hyperlipidemia (*p* < 0.001).

**Table 1:** Baseline characteristic of training, internal validation and external validation cohort.

Variables	Training cohort (n = 1791)	Internal validation cohort (n = 597)	External validation cohort (n = 907)
Male, n (%)	1258 (70.24%)	421 (70.52%)	636 (70.12%)
Age in years, median [Q1–Q3]	66 [58, 74]	67 [59, 74]	62 [53, 70]***
SBP (mmHg), median [Q1–Q3]	132 [122, 146]	133 [122, 147]	131 [121, 144]*
DBP (mmHg), median [Q1–Q3]	75 [68, 83]	75 [68, 83]	76 [69, 83]
HR (bpm), median [Q1–Q3]	74 [67, 80]	74 [67, 80]	74 [68, 80]
Laboratory indicators, median [Q1–Q3]			
FPG (mmol/L), mean ± SD	5.28 ± 0.64	5.29 ± 0.63	5.26 ± 0.64
TC (mmol/L)	3.63 [3.17, 4.20]	3.61 [3.10, 4.19]	3.42 [2.94, 4.00]***
TG (mmol/L)	1.19 [0.89, 1.63]	1.17 [0.87, 1.59]	1.24 [0.91, 1.67]
LDL-C (mmol/L)	1.84 [1.37, 2.35]	1.77 [1.32, 2.31]	2.13 [1.79, 2.57]***
HDL-C (mmol/L)	1.16 [0.99, 1.37]	1.16 [1.01, 1.37]	1.08 [0.94, 1.27]***
Scr (μmol/L)	78.60 [66.40, 92.30]	80.00 [67.00, 94.20]	69.00 [57.90, 80.70]***
History of alcohol intake, n (%)	490 (27.36%)	171 (28.64%)	233 (25.69%)
History of smoking, n (%)	571 (31.88%)	189 (31.66%)	254 (28.00%)*
Comorbidity, n (%)			
Hypertension	1291 (72.08%)	425 (71.19%)	567 (62.51%)***
Hyperlipemia	345 (19.26%)	109 (18.26%)	64 (7.06%)***
Family history of diabetes, n (%)	65 (3.63%)	21 (3.52%)	55 (6.06%)**
Statin, n (%)			
Use of statin	1240 (69.24%)	396 (66.33%)	762 (84.01%)***
High-intensity statin	33 (2.66%)	6 (1.52%)	12 (1.57%)
Non-high-intensity atorvastatin	766 (61.77%)	234 (59.09%)	459 (60.24%)
Non-high-intensity rosuvastatin	334 (26.94%)	111 (28.03%)	322 (42.26%)***
Pitavastatin	136 (10.97%)	49 (12.37%)	0 (0.00%)***
Psychotropic drugs, n (%)	242 (13.51%)	86 (14.41%)	232 (25.58%)***
ACEI, n (%)	219 (12.23%)	71 (11.89%)	134 (14.77%)
ARB, n (%)	563 (31.43%)	185 (30.99%)	200 (22.05%)***
β-B, n (%)	1012 (56.50%)	322 (53.94%)	223 (24.59%)***
CCB, n (%)	687 (38.36%)	212 (35.51%)	268 (29.55%)***
Diuretics, n (%)	536 (29.93%)	174 (29.15%)	237 (26.13%)*

SBP, systolic blood pressure; DBP, diastolic blood pressure; HR, heart rate; FPG, fasting plasma glucose, TC, total cholesterol; TG, triglyceride; LDL-C, low-density lipoprotein cholesterol; HDL-C, high-density lipoprotein cholesterol; Scr, serum creatinine; CVD, cardiovascular disease; ACEI, angiotensin-converting enzyme inhibitor; ARB, angiotensin receptor blocker; β-B, β-receptor blocking drugs; CCB, calcium channel blocker.

*p < 0.05, **p < 0.01, ***p < 0.001 versus training cohort.

The baseline characteristics of patients with and without NODAP within the training cohort are presented in Table S3. In comparison to patients without NODAP, patients with NODAP had elevated FPG and LDL-C levels, higher proportion of hypertension, decreased use of pitavastatin and increased use of ARB and diuretics (*p* < 0.001).

### Incidence of NODAP

After a median of 4.6 years of follow-up (IQR, 2.3–8.6 years), NODAP developed in 1,255 of 2,698 patients who underwent PCI for ACS from the training and validation cohorts, with a median of 57.2 months from PCI to the development of NODAP and an incidence rate of 46.52%. The cumulative incidence at 3, 6, 12, 24, and 36 months post-PCI was 0.93%, 2.04%, 5.60%, 12.02%, and 17.51%, respectively (Fig. S1).

### Variable selection

**Figure 2:**
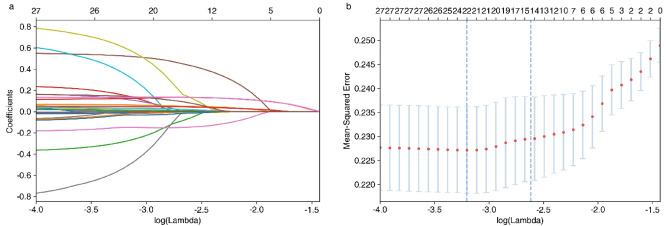
LASSO regression screening for NODAP variables. (**a**) LASSO model coefficient trendlines of the 27 risk factors for NODAP. The abscissa represents the optimal tuning parameter (lambda, λ), and the ordinate represents the regression coefficient. (**b**) Tuning parameter (lambda, λ) selection cross-validation error curve. The X-axis represents the optimal tuning parameter λ, and the Y-axis represents the binomial deviation. The longitudinal lines are drawn with 14–22 optimized non-zero coefficients obtained by tenfold cross-validation.

Within the training cohort, the 27 variables chosen in the previous stage were dimensionally reduced using LASSO regression (Fig. 2). Correlational cross-validation analysis of the variables resulted in 14–22 non-zero coefficient predictors potentially influencing the development of NODAP. The 14 potential risk factors identified were DBP, FPG level, LDL-C level, alcohol consumption history, smoking history, hypertension, hyperlipemia, family history of diabetes, use of statin, use of pitavastatin, use of non-high-intensity atorvastatin, use of psychotropic drugs, use of ARB, and use of diuretics. Table S4 shows the specific coefficients corresponding to the variables of lambda.1-se.

LASSO regression analysis of the 14 potential risk factors as independent variables, with NODAP as the dependent variable, was performed using multivariate logistic regression analysis. This identified the following 5 factors linked with the development of NODAP: FPG level, LDL-C level, hypertension, family history of diabetes and use of diuretics (Table 2), and there was no collinearity between any of the variables (Table S5).

**Table 2:** Multivariate logistic regression analysis of NODAP.

Variables	Coefficient	SE	Wald	p	OR	95%CI
(Intercept)	− 0.1326	0.049	7.184	0.007	0.876	0.795–0.965
FPG	0.389	0.051	57.369	< 0.001	1.476	1.334–1.632
LDL-C	0.155	0.050	9.636	0.002	1.167	1.059–1.287
Hypertension	0.325	0.051	40.360	< 0.001	1.384	1.252–1.531
Family history of diabetes	0.142	0.051	7.779	0.005	1.152	1.043–1.273
Diuretics	0.255	0.050	26.209	< 0.001	1.291	1.171–1.423

FPG, fasting plasma glucose; LDL-C, low-density lipoprotein cholesterol.

### Model establishment and web-based risk calculator development

**Figure 3:**
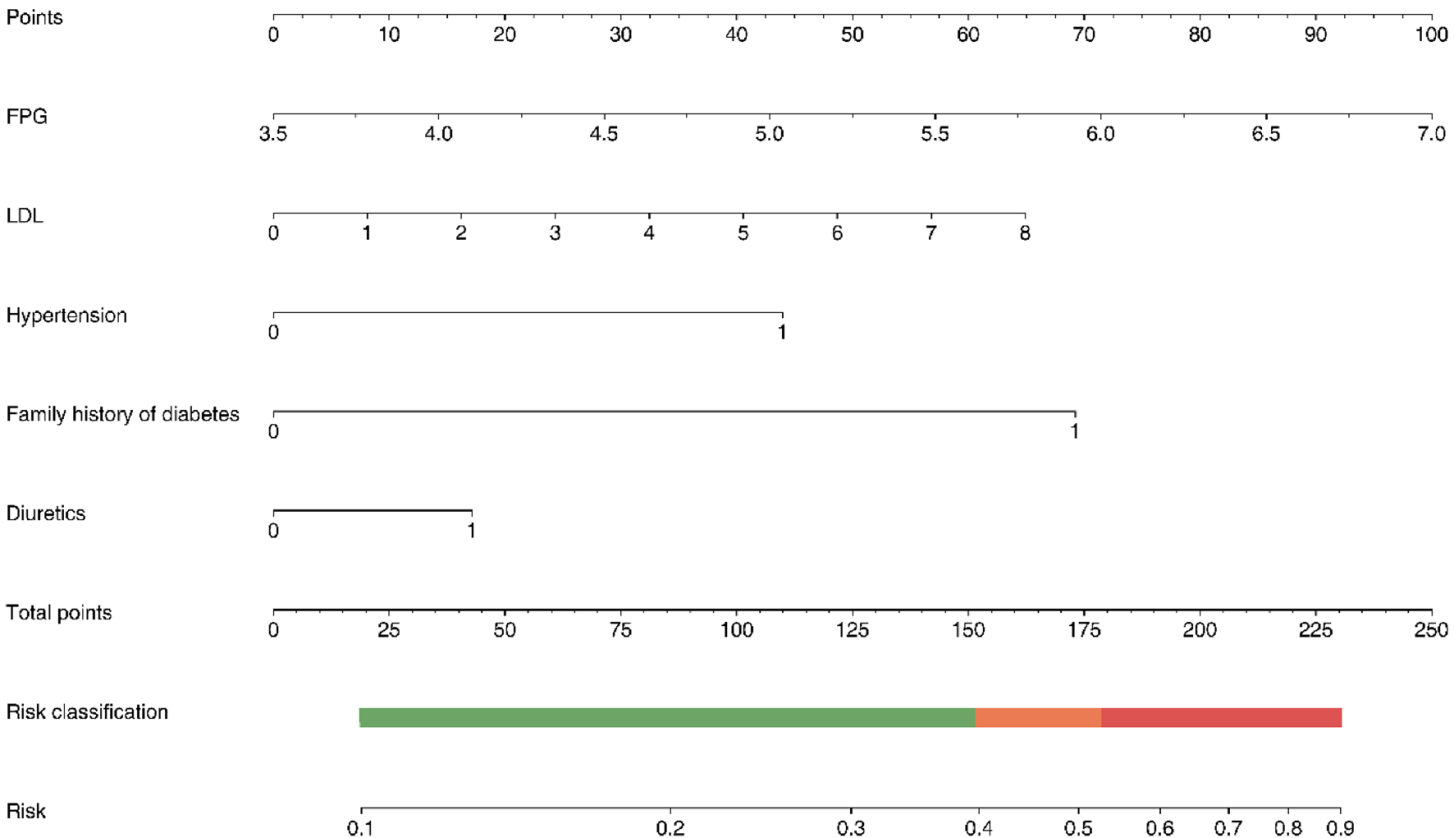
The Nomogram for NODAP. The length of each variable line segment in the nomogram reflects the influence of the variable on the occurrence of NODAP. The total score is the sum of the score values of each variable, and the scale value corresponding to the total score is the predicted risk probability of NODAP occurrence. Furthermore, according to the predicted risk, the risk group that the patient belongs to could be obtained. NODAP, New-Onset Diabetes After percutaneous coronary intervention; FPG, fasting plasma glucose; TC, total cholesterol; LDL-C, low-density lipoprotein cholesterol.

**Figure 4:**
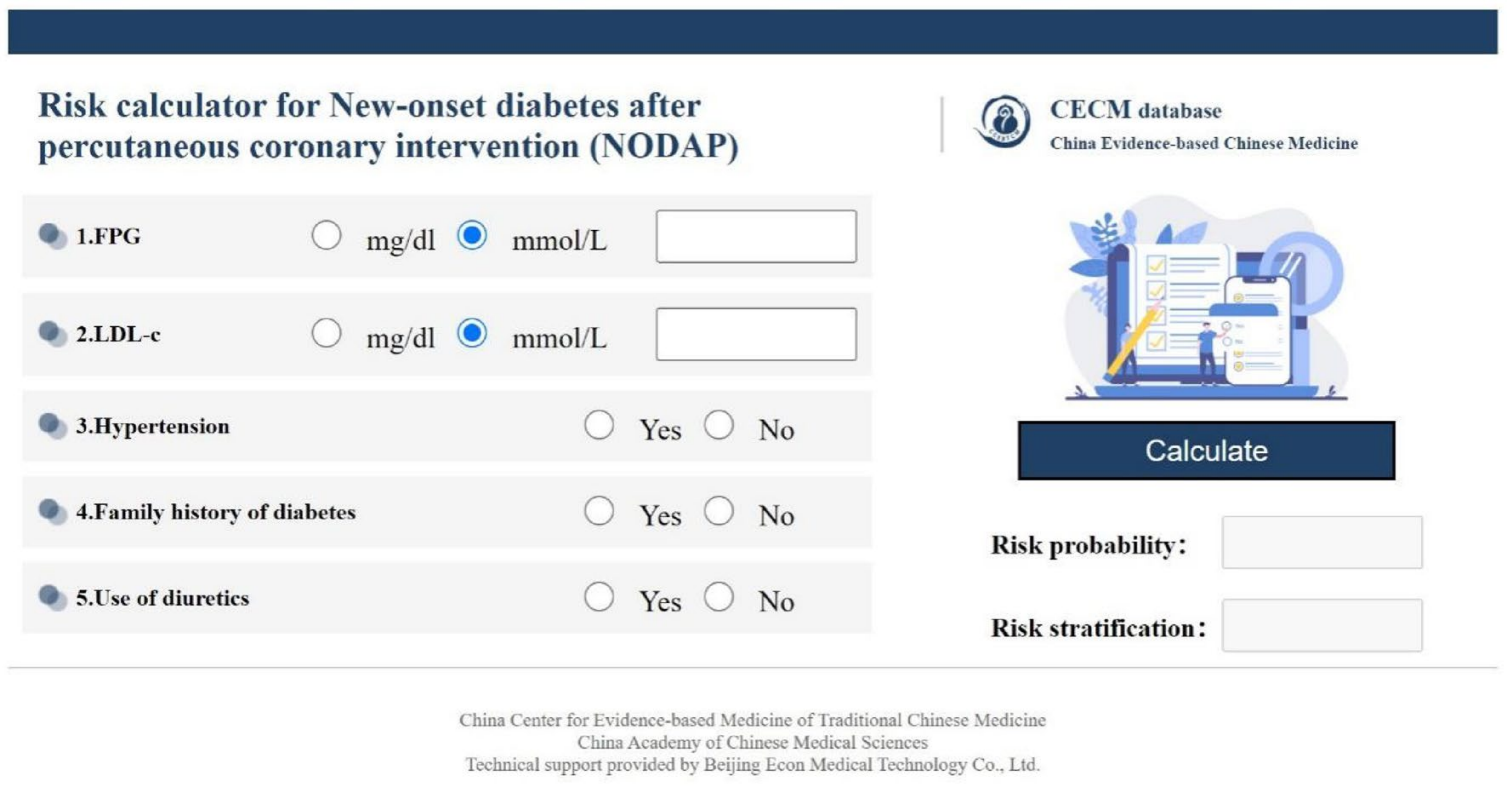
Webpage interface of the NODAP risk calculator. Users can input the variable results on the left side, and after clicking the “Calculate” button, the predicted risk probability and stratification can be displayed on the right side.

Within the training cohort, a NODAP nomogram was constructed with the 5 variables included in the multivariate logistic regression model: FPG level, LDL-C level, hypertension, family history of diabetes and use of diuretics (Fig. 3). The nomogram suggests that FPG level and family history of diabetes have the most significant impact on NODAP, followed by LDL-C level, hypertension, and use of diuretics. And the nomogram was then implemented into a web-based risk calculator (Fig. 4), which can allow clinicians directly derive the risk probability of NODAP after imputing results of the variables (https://mr.cscps.com.cn/wscoringtool/index.html).

### Model validation

**Figure 5:**
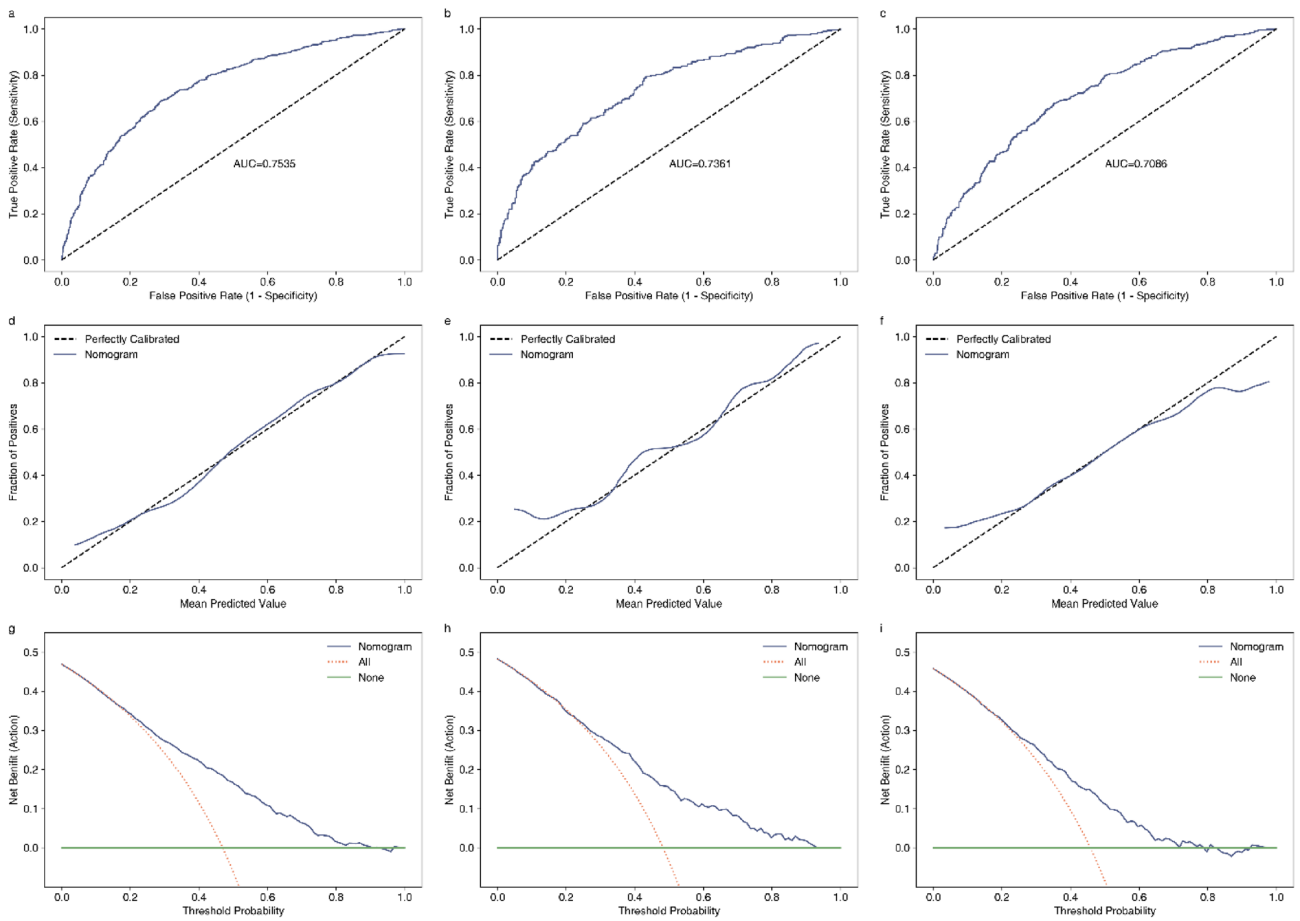
Validation efficacy of the nomogram for the NODAP in different cohorts. Analyze the distinguishing ability of the nomogram for NODAP in the development (**a)**, internal validation (**b**) and external validation cohorts (**c**); the calibration ability of in the development (**d**), internal validation (**e**) and external validation cohorts (**f**); the clinical benefits in the training (**g**), internal validation (**h**) and external validation cohorts (**i**).

The model was subjected to internal and external validation using the internal and external validation cohorts. The model's predictive performance was assessed in terms of discrimination, calibration, and clinical use. The AUCs were 0.7535, 0.7361, and 0.7086 for the training, internal validation, and external validation cohorts, respectively, indicating substantial model discrimination and satisfactory discriminative ability (Fig. 5a–c). The calibration curve, with the X-axis representing the predicted probability and the Y-axis representing the actual incidence probability, revealed good consistency between the predicted and observed values in the internal validation cohort and external validation cohort (Fig. 5d–f). And the results of the Hosmer–Lemeshow test in the training, internal validation, and external validation cohorts were χ^2^ = 41.684 (*p* = 0.060), χ^2^ = 31.753 (*p* = 0.331) and χ^2^ = 37.589 (*p* = 0.132), which also indicates the model fits well. The DCA demonstrated good clinical use of the model when the incidence probability ranged from 20 to 80% (Fig. 5g–i).

### Risk stratification

**Figure 6:**
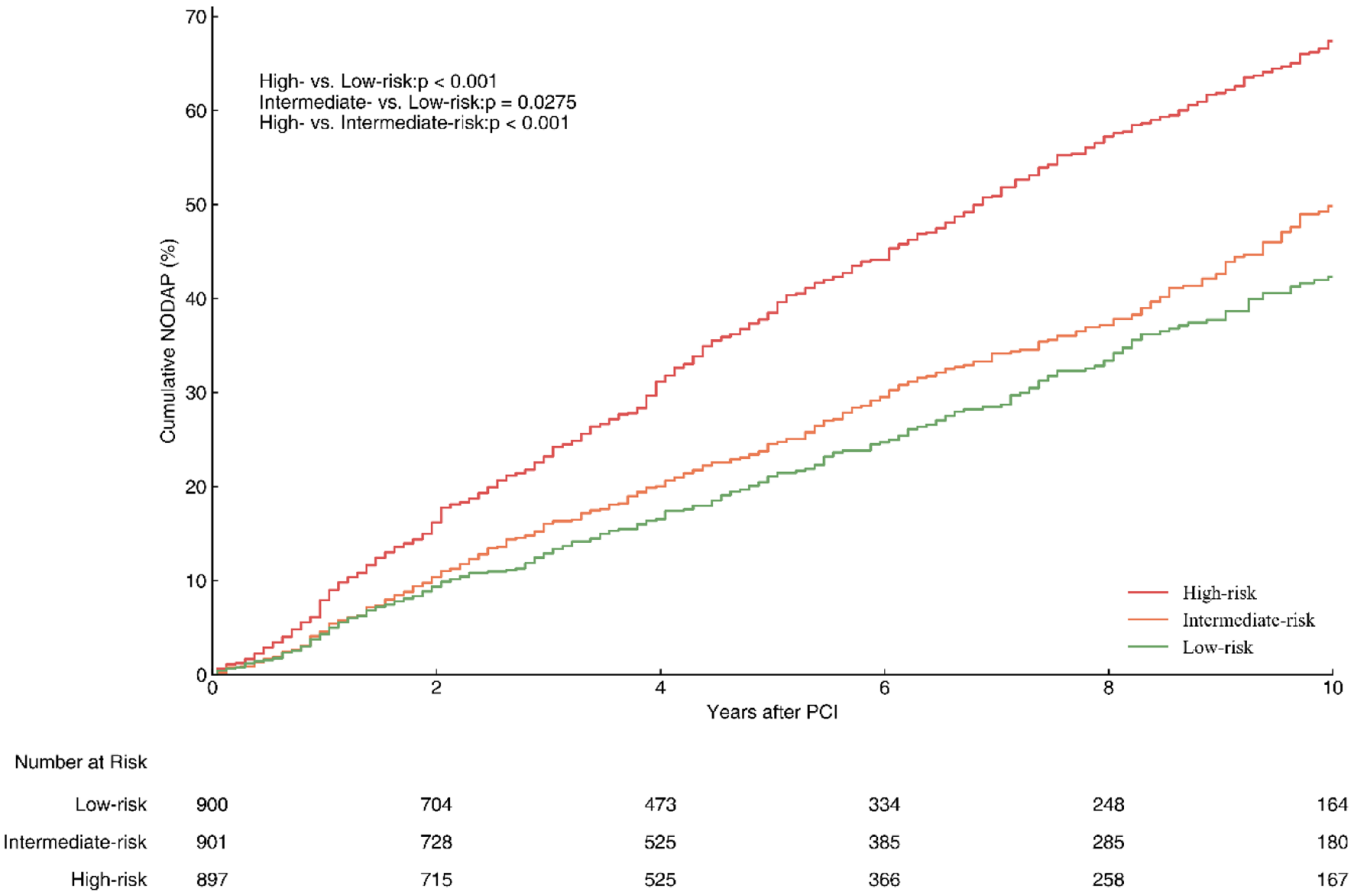
The cumulative incidence of NODAP for patients in the low-, intermediate-, and high-risk groups. NODAP, New-Onset Diabetes After percutaneous coronary intervention.

Beyond the establishment and validation of the nomogram, all patients in the training cohort were sorted based on their nomogram scores and classified into three NODAP risk groups (Fig. 3): low-risk (total points: 0–151.8; risk: 11.3–39.7%), moderate-risk (total points: 151.9–179.1; risk: 39.8–52.8%), and high-risk (total points: 179.2–230.3; risk: 52.9–90.0%). The cumulative incidence curve demonstrated that this risk stratification accurately differentiates patients with NODAP with different risk levels across the entire cohort (Fig. 6).

## Discussion

This study was conducted using medical record data from four centers in three provinces and cities of China and has thus pioneered the construction of a clinical prediction model for NODAP and implemented risk stratification. This study represents the first attempt to establish a nomogram and an online web-based risk calculator for predicting the risk of NODAP. At a median follow-up of 4.6 years (IQR, 2.3–8.6 years), the incidence of NODAP was established at 46.52%. The risk of NODAP can be appropriately evaluated through FPG level, LDL-C level, hypertension, family history of diabetes and use of diuretics. Our study is the first to report the long-term NODAP incidence rate in Chinese patients with ACS post-PCI, suggesting a high risk of new-onset diabetes that necessitates widespread attention. The prediction model we developed underwent internal and external validation with results, indicating good predictability.

Our predictive model revealed that the long-term incidence of NODAP exceeds the reported incidence of type 2 diabetes in the Chinese population^15^. Having a history of ACS might serve as an independent risk factor for new-onset diabetes, aligning with results drawn from other cohort study^16^. Nearly half of patients who undergo PCI must manage diabetes-related medical burden, thus underscoring the importance of appropriate and timely prediction of NODAP. Currently, there are more than 30 prediction models for the incidence of diabetes, each based on different populations^17^, with some models predicting the risk of new-onset diabetes in patients with coronary heart disease^8^. However, only one previous study has focused on the risk factors and incidence of NODAP in the Asian population^6^, with a lack of NODAP prediction models. Our study used rigorous methodology to tackle challenges such as missing data, model building, and internal and external validation, providing a clearer understanding of the groups at high-risk of NODAP.

Glycolipid metabolism levels were identified as significant risk factors for predicting the risk of NODAP, a conclusion that aligns with the findings of several prior studies that developed models to predict new diabetes onset^8,18,19^. Chun et al. determined through univariate and multivariate regression analyses that the risk factors for NODAP include FPG level ≥ 100 mg/dL, TG level ≥ 150 mg/dL, high body mass index (BMI), and high-intensity statins and that FPG is more influential than other factors^6^. FPG levels are significantly associated with the risk of diabetes^20^, and each 1 mg/dL increase in FPG leads to a 9% increase in the risk of diabetes, not only in patients with impaired fasting glucose, but also even in patients with normal FPG levels (90–99 mg/dL)^21^. 2 h postprandial glucose (2hPG) is also an indicator for detecting blood glucose levels, but studies have shown that the inclusion of 2hPG and insulin resistance in the prediction model did not significantly improve the predictive accuracy of the model^22,23^, and therefore NODAP risk prediction by incorporating a convenient and economical FPG is reliable and practical.

There is a consensus that higher LDL-C levels lead to an increased risk of cardiovascular events^24,25^, and LDL-C levels are the primary therapeutic target for lipid-lowering therapy in patients with coronary artery disease. Meanwhile, LDL-C has also been found to be associated with an increased risk of diabetes^26,27^, and the mechanism may be related to abnormal cholesterol metabolism affecting pancreatic β-cell membrane function and pancreatic cholesterol deposition, which leads to pancreatic β-cell dysfunction affecting glucose metabolism^28–30^. The prediction model in this study showed that LDL-C was also a very strong predictor of NODAP, and LDL-C levels were also significantly associated with the onset of NODAP, so controlling LDL-C levels in patients has the additional benefit of preventing and controlling the development of NODAP in addition to the reduced cardiovascular benefit^31^, but the specific mechanism of the effect of LDL-C on NODAP has yet to be investigated.

Family history of diabetes is a well-known risk factor for type 2 diabetes^32^, and the same results were obtained in this study for NODAP. Family history increases the risk of diabetes mellitus, which is thought to be mediated by a combination of genetic, environmental, and lifestyle pathways. Genetic susceptibility to diabetes mellitus has been demonstrated by several genome-wide association studies (GWAS), which have linked susceptibility location to pancreatic β-cell dysfunction, insulin resistance, and other factors^33^.

In addition, this study showed that NODAP risk was strongly associated with a history of hypertension rather than with transient values of BP. Hypertension and diabetes mellitus share multiple metabolic syndrome phenotypes including higher body mass index. abdominal obesity, hyperinsulinemia and hypertriglyceridemia^34,35^, and pathological processes such as dysregulation of renin–angiotensin–aldosterone system (RAAS), insulin resistance, and inflammation in hypertension can contribute to diabetes mellitus^36,37^. Therefore, our findings suggest that closer attention should be paid to glycemic indicators in patients undergoing PCI with hypertension.

Although a history of hypertension and a family history of diabetes are non-modifiable factors, evidence from several randomized controlled trials has demonstrated that the onset of diabetes can also be effectively delayed by improving lifestyle^38,39^. Therefore, our findings suggest that closer attention should be paid to the glycemic indexes of patients undergoing PCI with a family history of hypertension and diabetes, and consideration should be given to reducing the risk of NODAP through stricter lifestyle control.

Patients undergoing PCI are more likely to have hypertension and poor cardiac function and have more complex medications. Therefore, in our study we also considered the influence of prescribed medications, including antihypertensive drugs, psychotropic drugs, diuretics, and various types and intensities of statins on NODAP. We ultimately identified significant correlations between diuretics and NODAP, aligning with the outcomes of several clinical studies^40,41^. The mechanism by which diuretics affect glucose metabolism is mostly thought to be an indirect effect on insulin secretion due to diuretic-induced hypokalemia^42^, but its benefits in terms of reducing the occurrence of cardiovascular events are much greater^43^, and therefore it is still possible to use this drug for treatment with a combination of potassium-preserving diuretics or increased potassium supplementation and thus improvement of glucose metabolism, although the exact mechanism has not yet been fully elucidated^44^. This suggests that in patients at high risk of NODAP who require diuretic therapy, combined potassium-preserving measures may be considered to reduce the risk of morbidity. Previously, diuretics have also been found to have a dose-related effect on diabetes, and small doses of diuretics may not increase insulin resistance or insulin release^45,46^. We have not conducted further studies on the dose to be explored in the future. While previous studies on patients post-PCI have indicated that statin treatment increases the risk of NODAP by 27%^11^, and high-intensity statin treatment increases the risk of NODAP by 48%^6^, our study did not establish any association between statins or high-intensity statins and an increased risk of NODAP. Considering that the cardiovascular benefits of statin drugs considerably outweigh the adverse effects of new-onset diabetes^47,48^, statin drugs remain the primary choice for patients post-PCI.

In this study, we revealed that NODAP is a consequence of the combined effects of factors such as genetics, metabolism, and medication. We classified the risk of NODAP into low (11.3–39.7%), moderate (39.8–52.8%), and high (52.9–90.0%) risk. This stratification allows for improved formulation of post-PCI lifestyle and preventive antidiabetic regimens. Specifically, in the clinic, we can provide active NODAP preventive treatment to patients in advance based on their risk prediction results combined with diabetes prevention and treatment guidelines^49^. For example, if a patient is screened as intermediate risk, it is recommended that he/she should first control modifiable NODAP risk factors, including improving lifestyle, controlling glucose and lipid levels, etc. If he/she is screened as high risk, it is recommended that he/she should carry out intensive lifestyle interventions, including dietary control, exercise and avoiding the use of diuretics as much as possible. This study provides a precise risk calculator for patients with NODAP as well as prompts healthcare workers, especially those in the cardiovascular field, to pay attention to new-onset diabetes and underscores the urgent need for proactive NODAP prevention.

Despite its strengths, there are several limitations to this study: first, it relies on data from hospitals across three provinces and cities in China, with data from the chosen regions being both limited and unevenly spread, thus there may be selection bias. The long follow-up duration of the study may introduce potential attrition bias, and the precise onset date of diabetes could not be determined. Second, this research is a database-oriented retrospective study; therefore, there may be reporting inaccuracies or missing variables, such as height, weight and waist circumference reflecting obesity and type of ACS, number of diseased vessels, and number of stents implanted reflecting severity of the condition, as well as the inevitable recollection bias of the case data. And it was also not possible to obtain variable characteristics of lifestyle, dietary patterns, and frequency of exercise that may also affect NODAP. The lack of comprehensiveness of the variables covered also resulted in an inability to effectively compare with previous diabetes prediction models. Third, there are many differences between the training and validation cohort population characteristics, which may be related to the fact that the two parts of the cohort came from different provinces, different hospitals, and different severity of the disease, etc. More research centers, larger sample sizes, and more research variables need to be included to further externally validate the model efficacy. Additionally, the laboratory indicators used for diagnosing new-onset diabetes were dependent on glycated, fasting, and random blood glucose levels, which may not fully represent the long-term blood glucose control. Future research using oral glucose tolerance test results could potentially offer higher diagnostic sensitivity and precision. Lastly, we have only established a NODAP prediction and risk stratification system, and have not studied the prognosis of patients at different risks with different treatment modalities, nor have we been able to determine a causal relationship. Further studies are needed to investigate the effects of different management strategies on the long-term prognosis of patients undergoing PCI and the specific mechanisms linking these factors to NODAP.

## Conclusions

The incidence of NODAP is high and nearly 50% of post-PCI patients are at risk for future diabetes. This study developed and validated an intuitive, precise, and personalized risk prediction model and an online web-based risk calculator for NODAP, and a corresponding risk stratification system, showing that people with disorders of glucose and lipid metabolism (high FPG, LDL-C), hypertension, family history of diabetes, and those who are taking diuretics are more likely to develop NODAP, This nomogram can be used as a simple, practical clinical tool, assisting clinicians in accurately identifying the risk of new-onset diabetes following PCI surgery at an early stage. This model can be of great importance to provide prompt and efficient prevention of NODAP.

### Supplementary Information

Supplementary Information.

## Data Availability

All data generated or analyzed during this study are available from the corresponding authors upon reasonable request.

## Abbreviations

ACS: Acute coronary syndrome
ARB: Angiotensin receptor blocker
CECM: China evidence-based Chinese medicine
ROC: Receiver operating characteristic
DCA: Decision curve analysis
DM: Diabetes mellitus
FPG: Fasting plasma glucose
HDL-C: High-density lipoprotein cholesterol
LDL-C: Low-density lipoprotein cholesterol
MACE: Major adverse cardiac events
NODAP: New-onset diabetes after percutaneous coronary intervention
PCI: Percutaneous coronary intervention
TC: Total cholesterol
TG: Triglyceride

## References

[1] Bhatt DL (2018). Percutaneous coronary intervention in 2018. JAMA.

[2] Kosmidou I, Leon MB, Zhang Y (2020). Long-term outcomes in women and men following percutaneous coronary intervention. J. Am. Coll. Cardiol..

[3] Chen W-W, Chen J-Y, Li C-I (2020). Diabetes mellitus associated with an increased risk of percutaneous coronary intervention long-term adverse outcomes in Taiwan: A nationwide population-based cohort study. J. Diabetes Complicat..

[4] Lee CH, Choi S-W, Jun S-W (2020). Clinical impact of diabetes mellitus on 2-year clinical outcomes following PCI with second-generation drug-eluting stents; Landmark analysis findings from patient registry: Pooled analysis of the Korean multicenter drug-eluting stent registry. PLoS One.

[5] Konigstein M, Ben-Yehuda O, Smits PC (2018). Outcomes among diabetic patients undergoing percutaneous coronary intervention with contemporary drug-eluting stents: Analysis from the BIONICS randomized trial. JACC Cardiovasc. Interv..

[6] Chun KH, Im E, Kim BK (2017). Incidence, predictors, and clinical outcomes of new-onset diabetes mellitus after percutaneous coronary intervention with drug-eluting stent. J. Korean Med. Sci..

[7] Ertelt K, Brener SJ, Mehran R, Ben-Yehuda O, McAndrew T, Stone GW (2017). Comparison of outcomes and prognosis of patients with versus without newly diagnosed diabetes mellitus after primary percutaneous coronary intervention for ST-elevation myocardial infarction (the HORIZONS-AMI study). Am. J. Cardiol..

[8] Xu S, Scott CAB, Coleman RL, Tuomilehto J, Holman RR (2021). Predicting the risk of developing type 2 diabetes in Chinese people who have coronary heart disease and impaired glucose tolerance. J. Diabetes.

[9] Balachandran VP, Gonen M, Smith JJ, DeMatteo RP (2015). Nomograms in oncology—More than meets the eye. Lancet Oncol..

[10] Li Y, Cui J, Liu Y, Chen K, Huang L, Liu Y (2021). Development and validation of risk prediction model for new-onset diabetes after percutaneous coronary intervention (NODAP): A study protocol for a retrospective, multicenter analysis. Front. Cardiovasc. Med..

[11] Lin Z-F, Wang C-Y, Shen L-J, Hsiao F-Y, Lin Wu F-L (2016). Statin use and the risk for incident diabetes mellitus in patients with acute coronary syndrome after percutaneous coronary intervention: A population-based retrospective cohort study in Taiwan. Can. J. Diabetes.

[12] Yuan S, Larsson SC (2020). An atlas on risk factors for type 2 diabetes: A wide-angled Mendelian randomisation study. Diabetologia.

[13] Song DK, Hong YS, Sung Y-A, Lee H (2022). Association of serum creatinine levels and risk of type 2 diabetes mellitus in Korea: A case control study. BMC Endocr. Disord..

[14] Collins GS, Reitsma JB, Altman DG, Moons KGM (2015). Transparent reporting of a multivariable prediction model for individual prognosis or diagnosis (TRIPOD): The TRIPOD statement. BMJ.

[15] Society CD (2021). Guideline for the prevention and treatment of type 2 diabetes mellitus in China (2020 edition). Chin. J. Diabetes Mellit..

[16] Park CS, Chung WB, Choi YS (2015). Acute myocardial infarction is a risk factor for new onset diabetes in patients with coronary artery disease. PLoS One.

[17] Collins GS, Mallett S, Omar O, Yu L-M (2011). Developing risk prediction models for type 2 diabetes: A systematic review of methodology and reporting. BMC Med..

[18] Cai X, Zhu Q, Wu T (2020). Development and validation of a novel model for predicting the 5-year risk of type 2 diabetes in patients with hypertension: A retrospective cohort study. Biomed. Res. Int..

[19] Cai X-T, Ji L-W, Liu S-S, Wang M-R, Heizhati M, Li N-F (2021). Derivation and validation of a prediction model for predicting the 5-year incidence of type 2 diabetes in non-obese adults: A population-based cohort study. Diabetes Metab. Syndr. Obes..

[20] Sitnik D, Santos IS, Goulart AC (2016). Fasting glucose levels, incident diabetes, subclinical atherosclerosis and cardiovascular events in apparently healthy adults: A 12-year longitudinal study. Diabetes Vasc. Dis. Res..

[21] Munekawa C, Okada H, Hamaguchi M (2022). Fasting plasma glucose level in the range of 90–99 mg/dL and the risk of the onset of type 2 diabetes: Population-based Panasonic cohort study 2. J. Diabetes Investig..

[22] Wilson PWF, Meigs JB, Sullivan L (2007). Prediction of incident diabetes mellitus in middle-aged adults: The Framingham offspring study. Arch. Intern. Med..

[23] Gao WG, Dong YH, Pang ZC (2010). A simple Chinese risk score for undiagnose ddiabetes. Diabetic Med. J. Br. Diabetic Assoc..

[24] Navarese EP, Robinson JG, Kowalewski M (2018). Association between baseline LDL-C level and total and cardiovascular mortality after LDL-C lowering: A systematic review and meta-analysis. JAMA.

[25] Fulcher J, O’Connell R, Cholesterol Treatment Trialists’ (CTT) Collaboration (2015). Efficacy and safety of LDL-lowering therapy among men and women: Meta-analysis of individual data from 174,000 participants in 27 randomised trials. Lancet.

[26] Pan W, Sun W, Yang S (2020). LDL-C plays a causal role on T2DM: A Mendelian randomization analysis. Aging.

[27] Janghorbani M, Soltanian N, Amini M (2018). Low-density lipoprotein cholesterol and risk of type 2 diabetes: The Isfahan diabetes prevention study. Diabetes Metab. Syndr..

[28] Wei L, Wei M, Chen L (2021). Low-density lipoprotein cholesterol: High-density lipoprotein cholesterol ratio is associated with incident diabetes in Chinese adults—A retrospective cohort study. J. Diabetes Investig..

[29] Perego C, Da Dalt L, Pirillo A (2019). Cholesterol metabolism, pancreatic β-cell function and diabetes. Biochim. Biophys. Acta Mol. Basis Dis..

[30] Yao PM, Tabas I (2001). Free cholesterol loading of macrophages is associated with widespread mitochondrial dysfunction and activation of the mitochondrial apoptosis pathway. J. Biol. Chem..

[31] Huang J, Lin H, Wang S (2023). Association between serum LDL-C concentrations and risk of diabetes: A prospective cohort study. J. Diabetes.

[32] Hemminki K, Li X, Sundquist K, Sundquist J (2010). Familial risks for type 2 diabetes in Sweden. Diabetes Care.

[33] DIAbetes Genetics Replication and Meta-analysis (DIAGRAM) Consortium (2014). Genome-wide trans-ancestry meta-analysis provides insight into the genetic architecture of type 2 diabetes susceptibility. Nat. Genet..

[34] Tsimihodimos V, Gonzalez-Villalpando C, Meigs JB, Ferrannini E (2018). Hypertension and diabetes mellitus: Coprediction and time trajectories. Hypertension.

[35] Tsiachris D, Tsioufis C, Thomopoulos C (2011). New-onset diabetes and cardiovascular events in essential hypertensives: A 6-year follow-up study. Int. J. Cardiol..

[36] Aikens RC, Zhao W, Saleheen D (2017). Systolic blood pressure and risk of type 2 diabetes: A Mendelian randomization study. Diabetes.

[37] Mancusi C, Izzo R, di Gioia G, Losi MA, Barbato E, Morisco C (2020). Insulin resistance the hinge between hypertension and type 2 diabetes. High Blood Press. Cardiovasc. Prev..

[38] Knowler WC (2002). Reduction in the incidence of type 2 diabetes with lifestyle intervention or metformin. N. Engl. J. Med..

[39] Saito T, Watanabe M, Nishida J (2011). Lifestyle modification and prevention of type 2 diabetes in overweight Japanese with impaired fasting glucose levels: A randomized controlled trial. Arch. Intern. Med..

[40] Sabu SM, Seshadri S, Thunga G, Poojari PG, Acharya LD (2020). Assessment of association between antihypertensive drug use and occurrence of new-onset diabetes in south Indian patients. J. Pharm. Bioallied Sci..

[41] Huang C-Y, Ma T, Tien L (2013). A retrospective longitudinal cohort study of antihypertensive drug use and new-onset diabetes in Taiwanese patients. Biomed. Res. Int..

[42] Zillich AJ, Garg J, Basu S (2006). Thiazide diuretics, potassium, and the development of diabetes: A quantitative review. Hypertension.

[43] Scheen AJ (2018). Type 2 diabetes and thiazide diuretics. Curr. Diabetes Rep..

[44] Funder JW, Carey RM, Fardella C (2008). Case detection, diagnosis, and treatment of patients with primary aldosteronism: An endocrine society clinical practice guideline. J. Clin. Endocrinol. Metab..

[45] Bell DSH, Goncalves E (2021). Diabetogenic effects of cardioprotective drugs. Diabetes Obes. Metab..

[46] Mancia G, Grassi G, Zanchetti A (2006). New-onset diabetes and antihypertensive drugs. J. Hypertens..

[47] Wang K-L, Liu C-J, Chao T-F (2014). Risk of new-onset diabetes mellitus versus reduction in cardiovascular events with statin therapy. Am. J. Cardiol..

[48] Sattar N, Preiss D, Murray HM (2010). Statins and risk of incident diabetes: A collaborative meta-analysis of randomised statin trials. Lancet.

[49] ElSayed NA, Aleppo G, Aroda VR (2023). 3. Prevention or delay of type 2 diabetes and associated comorbidities: Standards of care in diabetes-2023. Diabetes Care.

