# Supplementary material for: Development, validation and visualization of a web-based nomogram for predicting risk of new-onset diabetes after percutaneous coronary intervention
Source: Sci Rep. 2024 Jun 13;14:13652. doi: 10.1038/s41598-024-64430-9 (PMC11176295; doi:10.1038/s41598-024-64430-9)
Supplement: Supplementary file 1 — Supplementary Information. [file 41598_2024_64430_MOESM1_ESM.docx]

**"Online Supplementary Materials"**

**Development, validation and visualization of a web-based nomogram for predicting risk of new-onset diabetes after percutaneous coronary intervention**

***Supplementary material***

Page 2: Figure S1

Page 3-4: Table S1

Page 5: Table S2

Page 6-7: Table S3

Page 8: Table S4

Page 9: Table S5

**Figure S1.** The cumulative incidence of NODAP


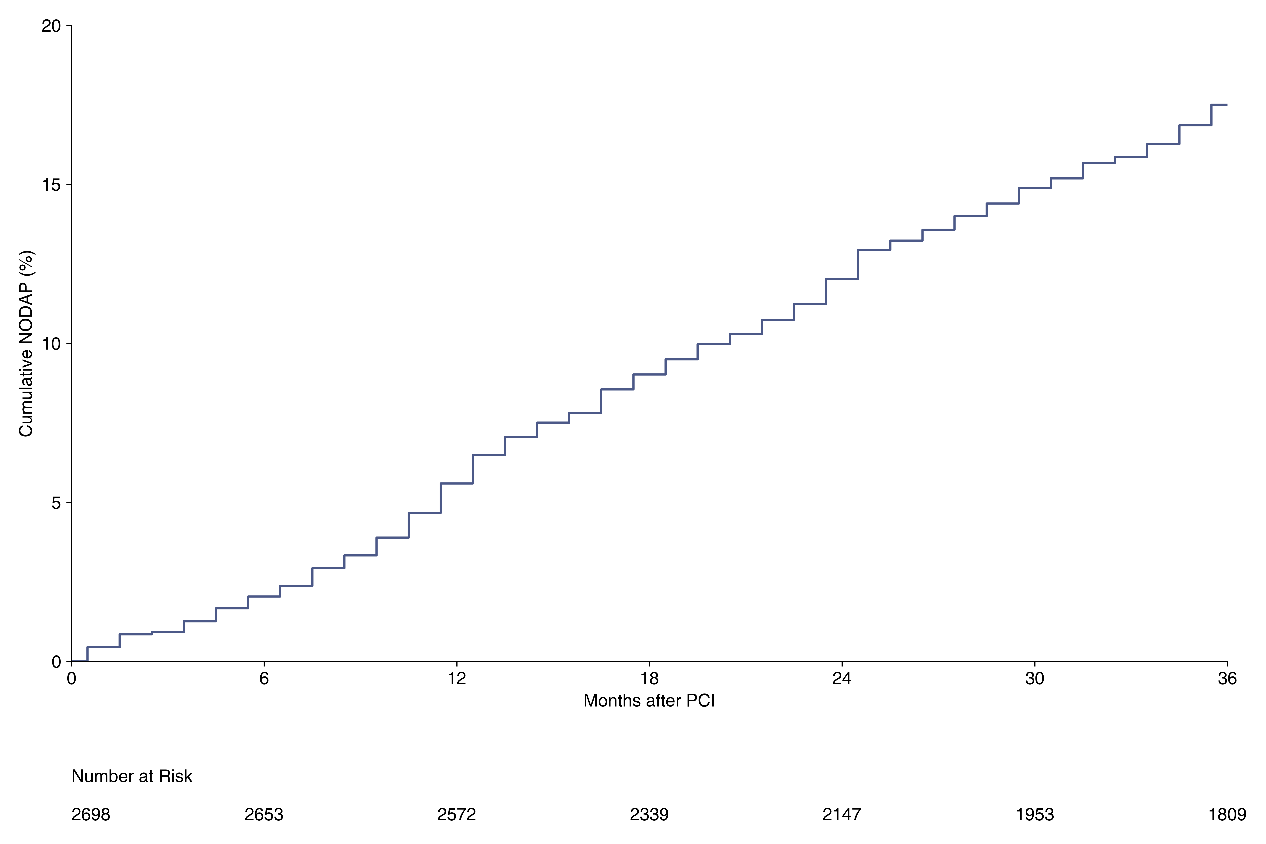


**Table S1.** Introduction of variables

| **Variables** | **Definition** |
| --- | --- |
| **Male, n（%）** | Male or female |
| **Age in years** | Age at the time of PCI |
| **SBP (mmHg)** | Admission Systolic BP at first hospitalization for PCI |
| **DBP (mmHg)** | Admission Diastolic BP at first hospitalization for PCI |
| **HR (bpm)** | Admission heart rate at first hospitalization for PCI |
| **Laboratory indicators** |  |
| **HbA1c (mmol/mol)** |  |
| **FPG (mmol/L)** |  |
| **TC (mmol/L)** | Laboratory results closest to admission date at first |
| **TG (mmol/L)** | admission for PCI |
| **LDL-C (mmol/L)** |  |
| **HDL-C (mmol/L)** |  |
| **Scr (μmol/L)** |  |
| **History of alcohol intake** | Drinking alcohol at least 1 time per week and cumulatively for ≥ 6 months |
| **History of smoking** | Smoking ≥ 1 cigarette per day and ≥ 6 months of cumulative smoking |
| **Comorbidity** |  |
| **Hypertension** | Past medical history clearly indicates a history of "hypertension" |
| **Hyperlipemia** | Past medical history clearly indicates a history of "hyperlipemia " |
| **Family history of diabetes** | Patient's first-degree relatives (parents, siblings, children) with diabetes |
| **Statin** |  |
| **Use of statin** | Long-term medical orders in the order system include "statin"; or the medical record specifies "statin" as a "current long-term oral medication" |
| **High-intensity statin** | Atorvastatin 40~80mg，Rosuvastatin 20mg |
| **Non-high-intensity atorvastatin** | Atorvastatin 10~20mg |
| **Non-high-intensity rosuvastatin** | Rosuvastatin 5~10mg |
| **Pitavastatin** | Long-term medical orders in the order system include " Pivastatin "; or the medical record specifies " Pivastatin " as a "current long-term oral medication" |
| **Psychotropic drugs** | Long-term medical orders in the order system include " diazepam, olanzapine, mirtazapine, haloperidol, citalopram hydrobromide, sertraline, oxazepam, zolpidem tartrate, zopiclone, eszopiclone"; or the medical record specifies the above drugs as a "current long-term oral medication" |
| **ACEI** | Long-term medical orders in the order system include "ACEI drugs"; or the medical record specifies the above drugs as a "current long-term oral medication" |
| **ARB** | Long-term medical orders in the order system include "ARB drugs"; or the medical record specifies the above drugs as a "current long-term oral medication" |
| **β-B** | Long-term medical orders in the order system include "β-B drugs"; or the medical record specifies the above drugs as a "current long-term oral medication" |
| **CCB** | Long-term medical orders in the order system include "CCB drugs"; or the medical record specifies the above drugs as a "current long-term oral medication" |
| **Diuretics** | Long-term medical orders in the order system include "Diuretics"; or the medical record specifies the above drugs as a "current long-term oral medication" |

PCI, percutaneous coronary intervention; SBP, systolic blood pressure; DBP, diastolic blood pressure; HR, heart rate; HbA1c, glycated hemoglobin; FPG, fasting plasma glucose; TC, total cholesterol; TG, triglyceride; LDL-C, low-density lipoprotein cholesterol; HDL-C, high-density lipoprotein cholesterol; Scr, serum creatinine; CVD, cardiovascular disease; ACEI, angiotensin-converting enzyme inhibitor; ARB, angiotensin receptor blocker; β-B, β-receptor blocking drugs; CCB, calcium channel blocker

**Table S2.** Measurement data missingness

| **Variables** | **N missing** | **Missingness(%)** |
| --- | --- | --- |
| **Gender** | 0 | 0.00 |
| **Age in years** | 0 | 0.00 |
| **SBP (mmHg)** | 21 | 0.78 |
| **DBP (mmHg)** | 25 | 0.93 |
| **HR (bpm)** | 43 | 1.59 |
| **Laboratory indicators** |  |  |
| **HbA1c (mmol/mol)** | 900 | 33.36 |
| **FPG (mmol/L)** | 728 | 26.98 |
| **TC (mmol/L)** | 448 | 16.60 |
| **TG (mmol/L)** | 448 | 16.60 |
| **LDL-C (mmol/L)** | 484 | 17.94 |
| **HDL-C (mmol/L)** | 450 | 16.68 |
| **Scr (μmol/L)** | 184 | 6.82 |

SBP, systolic blood pressure; DBP, diastolic blood pressure; HR, heart rate; HbA1c, glycated hemoglobin; FPG, fasting plasma glucose; TC, total cholesterol; TG, triglyceride; LDL-C, low-density lipoprotein cholesterol; HDL-C, high-density lipoprotein cholesterol; Scr, serum creatinine

**Table S3.** Baseline characteristic of NODAP and Non-NODAP in training cohort

| **Variables** | **NODAP**  **(n=840)** | **Non-NODAP**  **(n=951)** | ***p* value^a^** |
| --- | --- | --- | --- |
| **Male, n（%）** | 576(68.57%) | 682(71.71%) | 0.147 |
| **Age in years, median [Q1-Q3]** | 67(59, 74) | 65(58, 73) | 0.023 |
| **Systolic BP (mmHg), median [Q1-Q3]** | 134[124, 147] | 131[121, 145] | 0.007 |
| **Diastolic BP (mmHg), median [Q1-Q3]** | 74[68, 82] | 75[68, 83] | 0.057 |
| **Heart rate(bpm), median [Q1-Q3]** | 74[68, 81] | 72[67, 78] | 0.003 |
| **Laboratory indicators,**  **median [Q1-Q3]** |  |  |  |
| **FPG (mmol/L), mean±SD** | 5.39±0.70 | 5.17±0.57 | <0.001 |
| **TC (mmol/L)** | 3.63[3.11, 4.23] | 3.64[3.22, 4.17] | 0.559 |
| **TG (mmol/L)** | 1.26[0.90, 1.71] | 1.14[0.89, 1.56] | 0.003 |
| **LDL-C (mmol/L)** | 1.91[1.44, 2.42] | 1.78[1.32, 2.30] | <0.001 |
| **HDL-C (mmol/L)** | 1.17[0.98, 1.38] | 1.16[1.00, 1.37] | 0.898 |
| **Scr (μmol/L)** | 78.91[65.78, 92.80] | 78.50[66.95, 92.00] | 0.973 |
| **History of alcohol intake, n（%）** | 251(29.88%) | 239(25.13%) | 0.024 |
| **History of smoking, n（%）** | 277(32.98%) | 294(30.91%) | 0.350 |
| **Comorbidity, n（%）** |  |  |  |
| **Hypertension** | 672(80.00%) | 619(65.09%) | <0.001 |
| **Hyperlipemia** | 157(18.69%) | 188(19.77%) | 0.564 |
| **Family history of diabetes, n（%）** | 41(4.88%) | 24(2.52%) | 0.008 |
| **Statin, n（%）** |  |  |  |
| **Use of statin** | 584(69.52%) | 656(68.98%) | 0.803 |
| **High-intensity statin** | 15(2.57%) | 18(2.74%) | 0.848 |
| **Non-high-intensity atorvastatin** | 373(63.87%) | 393(59.91%) | 0.152 |
| **Non-high-intensity rosuvastatin** | 155(26.54%) | 179(27.29%) | 0.768 |
| **Pitavastatin** | 44(7.53%) | 92(14.02%) | <0.001 |
| **Psychotropic drugs, n（%）** | 132(15.71%) | 110(11.57%) | 0.010 |
| **ACEI, n（%）** | 102(12.14%) | 117(12.30%) | 0.918 |
| **ARB, n（%）** | 310(36.90%) | 253(26.60%) | <0.001 |
| **β-B, n（%）** | 496(59.05%) | 516(54.26%) | 0.041 |
| **CCB, n（%）** | 351(41.79%) | 336(35.33%) | 0.005 |
| **Diuretics, n（%）** | 308(36.67%) | 228(23.97%) | <0.001 |

NODAP, New-Onset Diabetes After percutaneous coronary intervention; FPG, fasting plasma glucose, TC, total cholesterol; TG, triglyceride; LDL-C, low-density lipoprotein cholesterol; HDL-C, high-density lipoprotein cholesterol; Scr, serum creatinine; CVD, cardiovascular disease; ACEI, angiotensin-converting enzyme inhibitor; ARB, angiotensin receptor blocker; β-B, β-receptor blocking drugs; CCB, calcium channel blocker

^a^ the difference between NODAP and Non-NODAP

**Table S4.** Coefficients and lambda.1-se value of the LASSO regression based on the training cohort

| **Factors** | **Coefficients** | **Lambda. 1-se** |
| --- | --- | --- |
| **SBP** | -0.0532 | 0.0024 |
| **FPG** | 0.4287 |  |
| **LDL-C** | 0.1230 |  |
| **History of alcohol intake** | 0.0486 |  |
| **Hypertension** | 0.0074 |  |
| **Hyperlipemia** | 0.1332 |  |
| **Family history of diabetes** | -0.0040 |  |
| **Use of pitavastatin** | 0.0767 |  |
| **Use of statin** | -0.1452 |  |
| **Use of psychotropic drugs** | -0.0094 |  |
| **Use of non-high-intensity atorvastatin** | 0.0175 |  |
| **Use of ARB** | -0.0148 |  |
| **Use of diuretics** | 0.0423 |  |

SBP, systolic blood pressure; FPG, fasting plasma glucose; LDL-C, low-density lipoprotein cholesterol; ARB, angiotensin receptor blocker

**Table S5.** Collinearity analysis of predictive model variables

| **Variables** | **Coefficient** | **t** | ***p*** | **tolerance** | **variance inflation factor** | ***F*（*p*）** |
| --- | --- | --- | --- | --- | --- | --- |
| **(Intercept)** | -0.1326 | -2.680 | 0.007 | - | - |  |
| **FPG** | 0.389 | 7.574 | <0.001 | 0.998 | 1.002 |  |
| **LDL-C** | 0.155 | 3.104 | 0.002 | 0.992 | 1.008 | 139.516 |
| **History of hypertension** | 0.325 | 6.353 | <0.001 | 0.969 | 1.032 | (＜0.001) |
| **Family history of diabetes** | 0.142 | 2.789 | 0.005 | 1.000 | 1.000 |  |
| **Diuretics** | 0.255 | 5.119 | <0.001 | 0.978 | 1.023 |  |

FPG, fasting plasma glucose; LDL-C, low-density lipoprotein cholesterol
